# Cas9-catalyzed DNA Cleavage Generates Staggered Ends: Evidence from Molecular Dynamics Simulations

**DOI:** 10.1038/srep37584

**Published:** 2016-11-22

**Authors:** Zhicheng Zuo, Jin Liu

**Affiliations:** 1 Department of Pharmaceutical Sciences, University of North Texas System College of Pharmacy, University of North Texas Health Science Center, Fort Worth, TX 76107, USA

## Abstract

The CRISPR-associated endonuclease Cas9 from *Streptococcus pyogenes* (spCas9) along with a single guide RNA (sgRNA) has emerged as a versatile toolbox for genome editing. Despite recent advances in the mechanism studies on spCas9-sgRNA-mediated double-stranded DNA (dsDNA) recognition and cleavage, it is still unclear how the catalytic Mg^2+^ ions induce the conformation changes toward the catalytic active state. It also remains controversial whether Cas9 generates blunt-ended or staggered-ended breaks with overhangs in the DNA. To investigate these issues, here we performed the first all-atom molecular dynamics simulations of the spCas9-sgRNA-dsDNA system with and without Mg^2+^ bound. The simulation results showed that binding of two Mg^2+^ ions at the RuvC domain active site could lead to structurally and energetically favorable coordination ready for the non-target DNA strand cleavage. Importantly, we demonstrated with our simulations that Cas9-catalyzed DNA cleavage produces 1-bp staggered ends rather than generally assumed blunt ends.

The **c**lustered **r**egularly **i**nterspaced **s**hort **p**alindromic **r**epeats (CRISPR)/CRISPR-associated (Cas) systems provide bacteria and archaea with adaptive immunity against invasive viruses and plasmids^1^,^2^,^3^,^4^. The Cas9 protein from *Streptococcus pyogenes* (spCas9), combined with a single guide RNA (sgRNA), a synthetic fusion of CRISPR RNA (crRNA) and trans-activating crRNA (tracrRNA), has been adapted to a most widely used toolbox for gene editing in various living cells and organisms, demonstrating great potential toward further therapeutics applications^5^,^6^,^7^,^8^.

The biochemical experiments have established that double-stranded DNA (dsDNA) recognition and cleavage by Cas9 strictly require the presence of a short protospacer-adjacent-motif (PAM) in the non-target, or non-complementary, DNA strand (ntDNA) and the complementarity of the target DNA strand (tDNA) to the 10–12 nucleotide (nt) PAM-proximal “seed” region in the guide RNA^6^,^9^. Recently solved crystal structures of spCas9 reveal a bilobed architecture comprising of an α-helical recognition (REC) lobe and a nuclease (NUC) lobe (Fig. 1A)^10^,^11^,^12^,^13^,^14^,^15^,^16^. The NUC lobe incorporates two Mg^2+^-dependent nuclease domains, dubbed as RuvC and HNH, which are responsible for cutting the ntDNA and tDNA, respectively (Fig. 1B,C)^6^,^9^. The RuvC domain features a typical ribonuclease H (RNase H) fold with four functionally essential residues, D10, E762, H983 and D986 (Fig. 1B)^6^,^12^, in line with a two-metal-ion catalysis mechanism^17^,^18^,^19^. In contrast, the HNH domain is characterized by a ββα-metal motif with three catalytic residues, D839, H840 and N863 (Fig. 1C)^6^,^10^,^12^, consistent with a one-meta-ion mechanism^17^,^18^,^19^.

A wealth of biochemical and structural information has made remarkable contributions to the mechanistic insights into RNA-guided DNA recognition and cleavage by Cas9. However, many details of the cleavage process, such as how the catalytic Mg^2+^ ions mediate the connections between Cas9 and DNA, remain elusive in this system^11^,^12^,^16^. The recent crystal structures show a closest distance of ~5.5 Å between the superimposed Mn^2+^ ions and the non-bridging oxygen atoms of the putative scissile phosphate on the ntDNA^11^,^16^, which is considerably larger than the typical Mg-O coordination distance of 2.1 Å for catalysis, indicating the crystalized conformation is inactive for catalysis. Intriguingly, the HNH domain assumes an inactive conformation in all the complex structures^10^,^11^,^12^,^13^,^14^,^15^,^16^, in which the shortest distance of the catalytic residue H840 to the opposite cleavable site on the DNA is around 14 Å, which is far beyond the range required for catalysis. To date, no active state conformation together with Mg^2+^ ion has been reported. We hypothesize that the catalytic Mg^2+^ ions induce the conformational change to an active state by facilitating the proximity of Cas9 active sites and DNA. Here, we used molecular dynamics simulations to search for the active state and to test our hypothesis.

Unlike HNH domain-mediated tDNA cleavage, determining the exact cleavage site on the ntDNA is complicated due to the additional 3′->5′ exonuclease activity of the RuvC domain^6^,^16^, especially when the protospacer 5′-end is used for direct sequencing analysis or radiolabeled for gel electrophoresis analysis^2^,^6^,^9^. Moreover, the target site detection methods, including direct sequencing and gel electrophoresis analysis, appear to exhibit limited resolution in terms of discrimination of 1-nt length^6^,^9^. Therefore, it is still controversial as to whether the spCas9 cuts the ntDNA at the phosphate 3 base pair (bp) upstream of the canonical 5′-NGG-3′ PAM motif (denoted as −3**P**) or 4 bp apart (−4**P**) (see Fig. 1D)^6^,^9^,^10^,^11^,^20^. This naturally elicits another interesting and critical question: Does Cas9 produce blunt-ended breaks or staggered-ended breaks with overhangs in the target dsDNA? Some studies reported that Cas9 cuts the ntDNA at −3**P** as the tDNA at +3 **P**, thereby leaving blunt ends^9^,^20^, whereas others argued that −4**P** is the ntDNA cleavage site, implying staggered ends generated by Cas9^10^,^11^. Meanwhile, there exists another view that Cas9 produces either blunt or 1-bp staggered ends^5^,^6^. For genome-editing, staggered-ended DNA is more controllable than the blunt-ended DNA because the DNA repair would only occur at the staggered end rather than at either end. To determine which type of ends generated by Cas9 is critical to understand the mechanism of non-homologous end joining (NHEJ)-based gene repair following the DNA cleavage^21^,^22^,^23^, hence shedding lights on strategy development to enhance precise genome engineering.

Molecular dynamics (MD) simulation has emerged as a powerful tool to explore bio-macromolecule dynamics in complementary with experimental data. To investigate the mechanisms underlying Cas9-catalyzed cleavage process, here, we present the first computational simulation study on the Cas9-sgRNA-dsDNA system at the atomic level to address the following two questions: 1) Could the Mg^2+^ ions bridge the distance gap between the active sites of Cas9 and ntDNA? 2) Where is the cleavage site on ntDNA? Our simulation results demonstrate that introduction of two Mg^2+^ ions at −4**P** remarkably induces conformation changes to bring the active site of the RuvC domain and ntDNA into proximity, forming structurally and energetically favorable coordination in between the catalytic residues and the scissile phosphate for cleavage reaction to occur. Thus, we evidence that Cas9 generates 1-bp 5′-staggered breaks in the dsDNA.

## Results

### Mg^2+^ Ions Induce Conformational Changes to Bridge the Distance Gap between the Active Site of the RuvC Domain and the ntDNA

**Figure 1:**
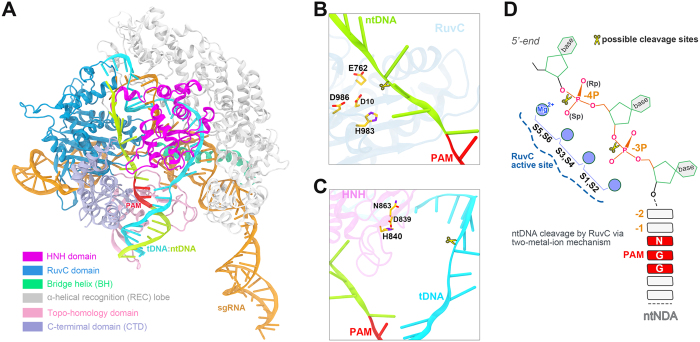
spCas9-sgRNA complexed with double strand DNA (dsDNA) substrate (**A**) and spCas9-catalyzed DNA hydrolysis (**B**–**D**). (**A**) Overall architecture of the ternary complex of spCas9, sgRNA and dsDNA. The domain organization of spCas9 is indicated in bottom-left corner. The sgRNA is colored orange, target DNA strand (tDNA) in Kelly green and non-target DNA strand (ntDNA) in cyan. (**B** and **C**) Close-up view of the catalytic center for RuvC (upper) and HNH (lower) nuclease domain. The catalytically essential residues are depicted in a stick model and colored by atom type (C, yellow; N, violet, O, red). (**D**) Schematic diagram showing the placement of Mg^2+^ pair at the interface of ntDNA and RuvC domain. **S1** and **S2** denote the replicated simulations in which the pair of Mg^2+^ were initially positioned around -3 phosphate (−3**P**), **S5** and **S6** around -4 phosphate (−4**P**), and **S3** and **S4** in between −3**P** and −4**P** (see Materials and Methods for more details). When labeling, the pro-Sp and pro-Rp oxygens of the phosphate group are abbreviated as Sp and Rp, respectively. In all panels, the 5′-NGG-3′ PAM trinucleotide in the ntDNA is highlighted in red. The putative cleavage site is denoted by a scissor shape.

To probe the effect of Mg^2+^ on conformational change, one Mg^2+^ ion was placed in the HNH domain on the basis of the one-metal ion catalysis mechanism (Fig. 1C), whereas two Mg^2+^ ions were positioned at the interface of the RuvC domain and the ntDNA at various positions according to the two-metal-ion mechanism (Fig. 1D)^17^,^18^,^19^. For simplicity, **S0** here represents the simulation in the absence of Mg^2+^; **S1**/**S2**, and **S5/S6** denote the repeated simulations in which the Mg^2+^ pair were positioned around −3**P** and around **−**4**P**, respectively, whereas **S3**/**S4** are the two simulations with the Mg^2+^ ions placed in between −3**P** and −4**P** (see Materials and Methods for details). Analysis of the root-mean-square deviations (RMSD) of the simulated systems revealed that the overall stability of the entire complex is largely determined by the sgRNA (Supplementary Fig. S1), as its long middle segment protrudes into the solvent, exhibiting high flexibility (Fig. 1 A). The Cas9 protein and the duplex DNA tended to reach equilibrium after dozens of nanoseconds, with RMSD fluctuating around 3 Å. The above observations are in line with a most recent study by Palermo *et al*.^24^. If not otherwise specified, all the following results were derived since 40 ns of the simulation trajectories.

**Figure 2:**
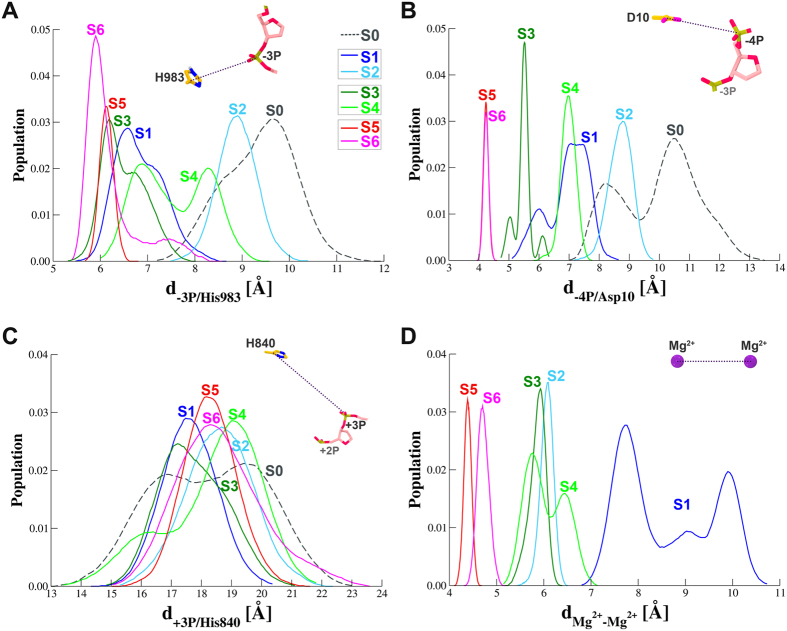
Distance distributions between the selected catalytic residues and the opposite phosphates on the target DNA (**A**–**C**) and between the two Mg^2+^ ions introduced to the RuvC domain (**D**) calculated from individual simulations. (**A**) Probability density for the distance between the C*γ* atom of His983 on the RuvC domain and the P atom of −3 phosphate on the ntDNA. (**B**) Probability density for the distance between the Cγ atom of Asp10 on the RuvC domain and the P atom of -4 phosphate on the ntDNA. (**C**) Probability density for the distance between the Cγ atom of His840 on the HNH domain and the P atom of +3 phosphate on the tDNA. (**D**) Probability density for the distance between the Mg^2+^ pair positioned at the RuvC domain. At the upper right of each panel, a schematic diagram is given to indicate the calculated distance.

We first monitored the proximity of the ntDNA and RuvC active center by measuring the distances between −3**P** and His983 (d_-3**P**/His983_) and between −4**P** and Asp10 (d_-4**P**/Asp10_) (Fig. 2A,B) for each trajectory. Without Mg^2+^ ions (**S0**), the distances between the ntDNA and the RuvC domain active residues peak at ~10 Å for both measurements (Fig. 2A,B). Compared to the control simulation (**S0**), addition of the Mg^2+^ ions obviously shifted the distance distributions of ntDNA and RuvC active center to smaller values in varying degrees regardless of the Mg^2+^ ions positions (**S1** to **S6**), indicating the presence of metal ions could indeed induce the conformational change toward the active state and close the distance gap between the ntDNA and the RuvC domain as previously postulated^11^,^12^,^16^ and as we hypothesized. Among all simulations, the double Mg^2+^ binding at the −4**P** in S**5** and S**6** leads to the largest reduction in d_-3**P**/His983_ (by ~4 Å) as well as in d_-4**P**/Asp10_ (by ~6 Å) when comparing the highest peaks with that of S**0** (Fig. 2A,B).

However, the HNH domain still adopted an inactive conformational state exhibiting no trend of moving close to the tDNA in all the simulations, as characterized by the distribution profiles of the distance of +3 **P** and His840 (d_+3**P**/His840_) that are largely overlaid with each other (Fig. 2C). Though the high intrinsic flexibility in the HNH domain^11^,^13^,^16^,^25^, our observation might result from the simulation time scale (hundreds of ns) far from sampling an activated state. More likely, in the context of dsDNA, the conformational switch of HNH domain is dependent on the completion of RuvC domain-catalyzed ntDNA cleavage accompanying by its product release^13^,^25^, which is beyond the scope of conventional MD simulation. In the following section, we focus on Mg^2+^-involved interactions with the RuvC domain and ntDNA.

### Mg^2+^ Ions Binding at −4P Lead to Active State Formation for ntDNA Cleavage

**Figure 3:**
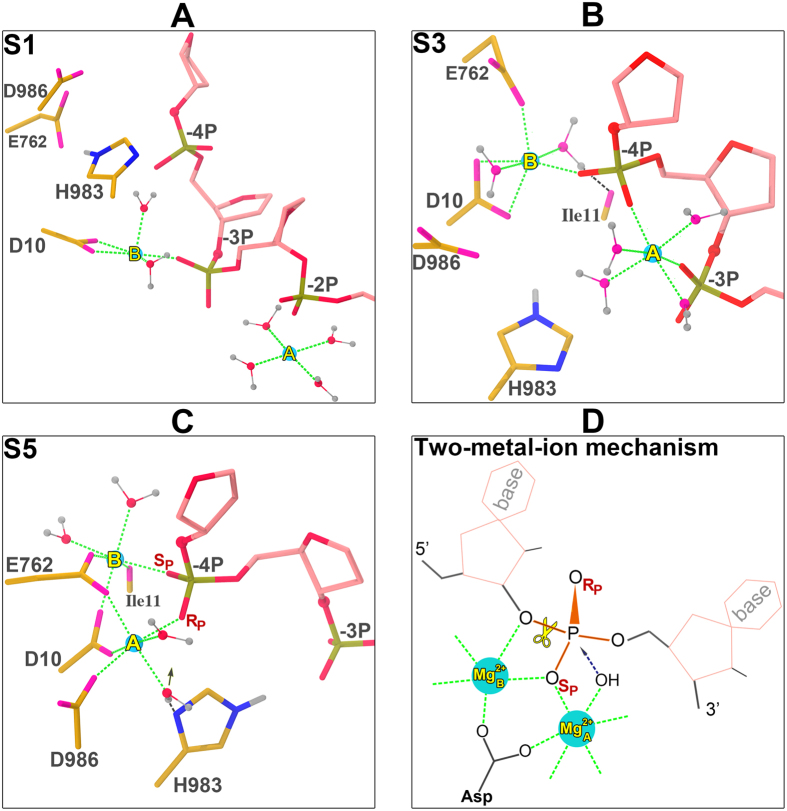
The representative coordination configurations involving the Mg^2+^ pair obtained from each set of simulations (**A**–**C**) and comparisons with the two-metal-ion catalysis by the RNase H (**D**). (**A**) The most-populated snapshot from S1. Note that the second largest cluster has a comparable population to the first one and the corresponding snapshot is present in Supplementary Fig. S2. (**B**) The most-populated snapshot from S3. (**C**) The most-populated snapshot from S5. (**D**) The schematic diagram of canonical two-metal-ion coordination by the RNase H. The RuvC and ntDNA residues are represented in stick model and colored by atom type, and the ligand water are shown as stick and ball style. The Mg^2+^ pair is illustrated as cyan spheres, with the one Mg^2+^ on the 3′ side of the ntDNA labeled “A” and the other one on the 5′ side labeled “B”. When labeling, the pro-Sp and pro-Rp oxygens of the phosphate group are abbreviated as Sp and Rp, respectively. The green dashed line indicates the coordination bond involving Mg^2+^, which is defined as atom-atom distance smaller than 2.2 Å. The black dashed line denotes the hydrogen bond. The potential nucleophilic water is attached by an arrow. The average coordination distances between Mg^2+^ and phosphate non-bridging oxygen, protein residue oxygen, water oxygen and protein residue nitrogen are 1.9 (0.1), 2.0 (0.1), 2.1 (0.1) and 2.2 (0.0) Å, respectively, from the simulations. See also the other snapshots for S2, S4 and S6 in Supplementary Fig. S3.

Further inspection of the simulation trajectories revealed distinct binding poses of the Mg^2+^ pair at the interface of RuvC domain and ntDNA. In **S1** and **S2**, the Mg^2+^ ion A (the one on the 3′ side of ntDNA) moves toward the −2**P** from its initial location at −3**P** (Fig. 1D, Fig. 3A and Supplementary Fig. S3A). In **S3** and **S4,** the Mg^2+^ B (the one on the 5′ side of ntDNA) departs from the middle position in between −3**P** and −4**P**, entering the negatively charged center enclosed by Asp10, E762 and D986 or −4**P** (Fig. 1D, Fig. 3B and Supplementary Fig. S3B). Positioning Mg^2+^ at either −3**P** (**S1/S2**) or in between −3**P** and −4**P** (**S3/S4**) all led to the Mg-Mg distance larger than 6 Å (Fig. 2D). In **S5** and **S6**, the Mg^2+^ pair stays around −4**P** (Fig. 1D, Fig. 3C and Supplementary Fig. S3C), resulting in the lowest Mg-Mg separation distance (4.3~4.7 Å) among all simulations, which is comparable to the inter-Mg^2+^ distance (4.1 Å) from the experimental and theoretical studies on another two-metal-ion catalytic system with two Mg^2+^ trapped in between the *Bacillus halodurans* ribonuclease H (RNase H) and an RNA/DNA hybrid (Fig. 3D and Supplementary Fig. S3D)^17^,^26^,^27^,^28^.

In the framework of two-metal-ion mechanism, the two metal ions are coordinated by an invariable Asp (that may be a phosphate in ribozymes) and the scissile phosphate^17^,^18^,^19^. Among all the simulations, only the coordination composition and geometry captured in **S5** and **S6** (Fig. 3C; Supplementary Fig. S3C), in which the conserved Asp10 and the −4**P** contribute two coordination ligands to the double Mg^2+^, respectively, closely match those observed in the prototype RNase H system (Fig. 3D and Supplementary Fig. S3D). Unlike the other simulations (**S1**/**S3** in Fig. 3A,B and **S2**/**S4** in Supplementary Fig. S3A,B), in which Mg^2+^ ions only coordinated with one or two catalytic residues, all three acid catalytic residues (Asp10, E762 and Asp986) are involved in coordination with the two metal ions in **S5** and **S6**. Specifically, it is interesting to note that despite the similarity in the coordination configuration, the general base His983 in S**6** is directly engaged to the Mg^2+^ A (Supplementary Fig. 3C), whereas in S**5** it is hydrogen-bonded to a water molecule that provides a ligand to the Mg^2+^ A coordination (Fig. S3C).

Taken together, the observations uncover formation of an active state for ntDNA cleavage stemming from the binding of two Mg^2+^ at –4 **P**. Yet it should be mentioned that compared with the canonical two-mental-ion coordination in the RNase H system, there exists a noticeable difference in terms of the scissile phosphate-mediated coordination with the two Mg^2+^. Both in **S5** and **S6**, the two non-bridging oxygens (pro-Sp and pro-Rp) of −4**P** together form a bidentate coordination with the double Mg^2+^ ions (Fig. 3C and Supplementary Fig. S3C), whereas in the RNase H-substrate complex, only the pro-Sp oxygen is implicated (Fig. 3D and Supplementary Fig. S3D). This may account for why the separation of the two Mg^2+^ (Fig. 2D) and that of the Mg^2+^ B and the leaving group 3′-O in **S5** and **S6** (Fig. 3C and Supplementary Fig. S3C) are slightly larger than those observed in the crystal structure of RNase H complex (Fig. 3D and Supplementary Fig. S3D).

### The Double Mg^2+^ Binding at −4P is Energetically More Favorable than Binding at Other Positions

To provide a quantitative view of the preference of the two Mg^2+^ ions for the scissile phosphate, we estimated the binding free energies of the Mg^2+^ pair at various positions via the end-point MM-GBSA approach^29^,^30^,^31^. Compared to the alternative MM-PBSA, MM-GBSA is computationally more efficient and has demonstrated the advantage in ranking the affinities of a series of ligands^32^,^33^,^34^. Given that water molecules play an important role in coordinating the catalytic ions (Fig. 3 and Supplementary Fig. S3), we consistently retained four water molecules that are closest to the two Mg^2+^ as part of receptor in the trajectories for MM-GBSA calculations (see Materials and Methods for details). The results showed that the binding of the Mg^2+^ pair at −4**P** in **S5** and **S6** is more stable than those at −3**P** or in between −3**P** and −4**P** in other simulations (**S1**–**S4**) (Table 1). Moreover, the binding free energy values of the set of parallel simulations are comparable in magnitude, indicating similar stability for the two coordination geometries captured in **S5** and **S6** (Fig. 3C and Supplementary Fig. S3C). We also calculated the non-bonded interaction energy of the Mg^2+^ pair with all surrounding residues and water and found a similar trend to that of MM-GBSA (Supplementary Table S1).

**Figure 4:**
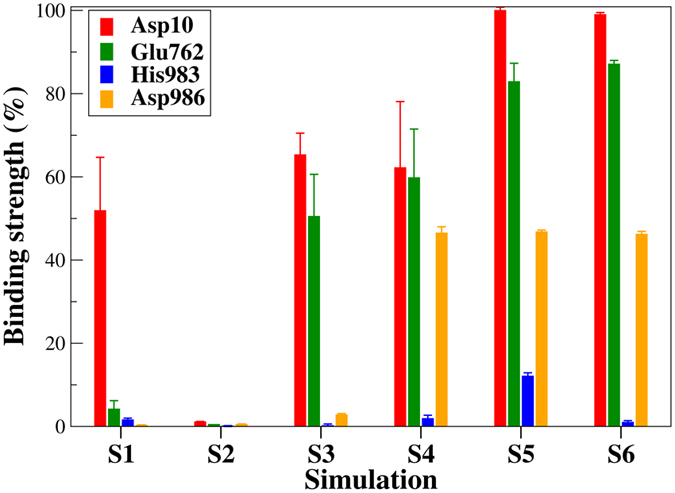
Relative binding strength for the RuvC catalytic residues calculated from respective sets of simulations via the MM-GBSA approach. Note that among all the simulations, the Asp10 in S5 gives the largest energetic contribution and hence is indicated 100% binding strength in the bar plot to which other residues are compared.

The biochemical experiments have evidenced the functional importance of four residues (viz. Asp10, Glu762, His983 and Asp986) at the RuvC active center for Cas9 catalytic activity^6^,^12^. We further performed per-residue free energy decomposition on these four residues to determine the contributions of individual catalytic residues to the binding of the metal ions. In **S2**, all of the four active residues hardly contribute to the binding of the two Mg^2+^, while Asp10 is the sole major contributor in **S1** (Fig. 4). Compared to **S3**, Asp986 in **S4** shows much stronger binding affinity (Fig. 4), accounting for the marked difference in binding free energy for the two simulations (Table 1). Notably, the two acid residues, Asp10 and Glu762 in **S5**/**S6** make much larger contributions to the binding than in other simulations (Fig. 4), again suggesting most favorable binding of the Mg^2+^ at –4 **P** (Table 1). In contrast, His983 contributes marginally to stabilizing Mg^2+^, which is consistent with its major role as a general base activating the nucleophile^17^,^18^,^19^. The per-residue contribution analyses here are in line with the above structural observations (Fig. 3 and Supplementary Fig. S3).

## Discussion and Conclusions

Several crystal structures of spCas9 have been solved, however, none of these structures assume the catalytic active state. One of the reasons is that to crystalize the ternary complex of spCas9-sgRNA-dsDNA, cleavage has to be prevented by substitution of catalytic residues and/or chelation of metal ions so that the systems do not support catalysis^12^,^16^. The use of partial ntDNA (cleaved product) for complex crystallization may also impair two metal ion binding^10^,^14^,^15^. In the complex structure with PAM-containing partial DNA duplex (PDB code: 4UN3), for example, only a single Mg^2+^ is non-specially bound at the RuvC active site, forming an incomplete coordination due to lack of the involvement of phosphate group (Supplementary Fig. S4A)^10^. It is interesting to note that the ion A of the Mg^2+^ pair in our simulations spatially overlaps with that one in 4UN3 (Supplementary Fig. S4A). In the apo-Cas9 without sgRNA and dsDNA (PDB code: 4CMQ), two Mn^2+^ ions instead of Mg^2+^ were found at the RuvC active site but only at a concentration much higher than physiologically relevant^11^ (Supplementary Fig. S4B). Here, for the first time, we determined the catalytic active state for the RuvC domain and the ntDNA by scanning the possible scissile phosphates on the ntDNA with a pair of Mg^2+^. The presence of Mg^2+^ ions at −4**P** remarkably drive the RuvC domain active center to the opposite phosphate backbone (Fig. 2), leading to a reactant-like coordination primed for cleavage (Fig. 3 and Supplementary Fig. S3). We demonstrated that beyond its catalytic role, the second role of Mg^2+^ ions is to lead the inactive conformation of the RuvC domain and the ntDNA toward the active state for catalysis. Our results add a piece of evidence corroborating the argument that acquisition of metal ions could strength specific substrate recognition and association, as observed in many polymerases and nucleases^17^,^18^,^19^.

From the twin Mg^2+^ coordination fashions and the calculated binding free energies in different sets of simulations, we concluded that it is very unlikely for the RuvC domain to cut the ntDNA at −3**P** due to obvious disobedience with the two-metal-ion mechanism that entails joint coordination of the Mg^2+^ pair by the scissile phosphate and a conserved Asp (Fig. 3 and Supplementary Fig. S3), as well as the unfavorable binding free energy comparing to other binding conformations (Table 1). Rather, we argue that −4**P** is the ntDNA cleavage site based on the formation of structurally and energetically favorable coordination, which is supported by the enzymatic footprinting experiments^11^, and also consistent with the structural observation showing the acidic residue cluster in the RuvC domain active center is spatially closer to −4**P** than −3**P**^16^. To further enhance the reliability of the conclusion, we additionally performed four shorter simulations (4 × 60 ns) starting with randomly assigned velocities for each of the three binding configurations (i.e. Mg^2+^ pair placed at −3**P**, −3**P**/−4**P** and −4**P**, respectively), and found very similar results with respect to the two longer simulations here (2 × 200 or 2 × 300 ns) (see full details in the Supplementary Information). Since the HNH domain-mediated tDNA cleavage has been more unambiguously identified as occurring at +3**P**^9^,^11^, we rationalize 1-bp 5′-staggered ends initially generated by Cas9 other than generally assumed blunt ends^9^,^20^. This staggered end may be further extended to a length of interest by rationally exploiting the additional 3′->5′ exonuclease-trimming activity in the RuvC domain^6^,^16^, which could be particularly advantageous for facilitating non-homologous end joining (NHEJ)-mediated gene insertion into the mammalian genome^21^,^22^,^23^. Considering that the small molecules that enhance NHEJ activities have been demonstrated to enhance the CRISPR/Cas genome editing^35^,^36^, we speculate that mutations or small molecules that could modulate the cleavage site to make the 1-bp staggered end more “stagger” may also help to enhance the CRISPR/Cas9 genome editing.

In this report, we did not address the questions on the HNH domain. For example, what triggers the HNH domain to convert into an active conformation? How does the Mg^2+^ ion bridge the distance gap between the HNH domain and the tDNA? These intriguing questions will be investigated in our future studies. We also noticed the derived two-metal-ion coordination geometries are somewhat deviated from that by the RNase H (Fig. 3C,D and Supplementary Fig. S3C,D), though this does not qualitatively influence the conclusions drawn here. We suspect that the discrepancy is related to the additive force field, but we cannot rule out the possibility that two-metal-ion catalysis mechanism of Cas9 is slightly different from that of RNase H. These issues will be addressed in our future studies by using the polarizable force fields in the MD simulations and using the quantum mechanical/molecular mechanical (QM/MM) approaches to explore the free energy surface for catalysis reactions^28^.

In summary, our study indicates that binding of two catalytic Mg^2+^ ions at the RuvC domain catalytic center facilitates the formation of an active state for ntDNA cleavage, and, importantly, that Cas9-catalyzed target DNA cleavage produces 1-bp staggered ends rather than the blunt ends. The staggered ends have more advantages than the blunt ends in genome editing because the staggered ends help the DNA insertion in the right direction, making genome editing more efficient and more specific. The short staggered ends revealed in this study shed lights on further improvement of CRISPR/Cas9 technology to increase its efficiency and specificity.

## Materials and Methods

### System Setup

The initial coordinates of Cas9-sgRNA-dsDNA were taken from the Protein Data Bank (PDB code: 5F9R^16^). Compared to other DNA-bound crystal structures^10^,^12^,^14^,^15^, 5F9R has no missing residues and contains no mutations. Of particular note is that the dsDNA in 5F9R is trapped in a pre-cleavage state with an intact non-target strand, which is especially suitable for this study. However, catalytic metal ions are excluded from all the available DNA-bound crystal structures. Based on the two-metal-ion catalysis mechanism proposed for the RuvC domain^11^,^12^,^16^, two Mg^2+^ ions were positioned at 5′- and 3′-sides of the pre-Sp oxygen of the -3 phosphate (−3**P**) or −4 phosphate (−4**P**) (Fig. 1D and Table 2), respectively, by reference to the crystal structure of Mg^2+^-bound RNase H in complex with an RNA/DNA hybrid (PDB code: 1ZBI)^17^,^26^,^27^. Additionally, the intermediate Mg^2+^ locations between −3**P** and −4**P** were also taken into account, in which one Mg^2+^ ion was placed close to 5′-side of the pro-Sp oxygen of −3**P**, and the other to 3′-side of the pro-Sp oxygen of −4**P** (Fig. 1D and Table 2). Given that other sites except −3**P** and −4**P** are evidently too far from RuvC catalytic center, we did not introduce Mg^2+^ ions therein (Fig. 1B,D). Meanwhile, we added one Mg^2+^ ion to the HNH domain following the one-metal-ion mechanism^17^,^18^,^19^. Note that in this crystal structure, the HNH domain active site is still considerably apart from the cleavable site on the tDNA as reflected by a distance of 17.25 Å between the His840 Cγ atom and the P atom of +3**P**, while the corresponding value is far higher in other target-bound structures (32~35 Å)^10^,^12^,^14^,^15^. Each Mg^2+^-bound complex above was solvated with TIP3P water molecules with a thickness of 13.5 Å, leading to a periodic boundary box of 139 × 124 × 187 Å^3^. To mimic the reaction buffer^6^,^9^, extra 16 Mg^2+^ were added into the water box to generate a concentration of 10 mM, and the ionic strength of KCl was set to 100 mM. As a control, the original system without Mg^2+^ was also simulated (Table 2). The total atoms of each system add up to ~282,000.

### Molecular Dynamics Simulations

All the simulations were performed by the AMBER15 *pmemd* engine with GPU acceleration^37^. The amber ff14SB force fields^38^ were used to describe the atomic interactions involving the protein and nucleic acids, and the recently developed ion parameter sets optimized in TIP3P water^39^,^40^ were selected for the mono- and divalent ions. It is noted that none of the available non-bonded models for Mg^2+^ ion is able to reproduce various experimental properties simultaneously; the set of Mg^2+^ parameters here represent the best possible compromise targeting the experimental Mg-O distance, hydration free energy as well as coordination number^40^. The non-bonded interactions were truncated at 10 Å, and the long-range electrostatics were treated via the particle mesh Eward summation (PME) method^41^ with a grid spacing of 1 Å. The covalent bonds involving hydrogen atoms were constrained employing the SHAKE algorithm^42^. Each simulation system was first subjected to a thorough energy minimization with the solute backbone atoms restrained, followed by slow heating from 0 K to 310.15 K and 10-ns equilibration in the isothermal-isochoric (NVT) ensemble with a time step of 1 fs. The production simulations were conducted under the isothermal-isobaric (NpT) conditions using a time step of 2 fs and were extended to at least 200 ns (Table 2), in which the temperature was maintained at 310.15 K through the Langevin thermostat and the pressure was controlled at 1.013 bar by the Monte Carlo barostat. The trajectory snapshots were saved every 10 ps. Except for the control system, two long parallel simulations (2 × 200 or 2 × 300 ns) and four additional short simulations (4 × 60 ns) with different random seeds were performed for the remaining ones (Table 2; see also Supplementary Information).

### Cluster Analysis

The representative (i.e. most-populated) configurations involving the double-Mg^2+^ coordination were determined through the cluster analysis with the package VMD^43^. For each simulation trajectory, the structure ensemble of snapshots since 40 ns was grouped into four clusters by trying varying root-mean-square deviation (RMSD) cutoffs (0.6–1.4 Å), after structural alignment on the reaction interface comprising the four catalytic residues (D10, E762, H983 and D986) on the RuvC domain, the backbone of nucleotides -3 and -4 on the ntDNA and the Mg^2+^ pair lying between them. In the final trials, the first two clusters were found to account for >90% of total population and the snapshot closest to the centroid of largest structural ensemble was extracted for comparative analysis.

### Binding Free Energy Calculations and Per-residue Energy Decomposition

The binding stability of the Mg^2+^ pair at respective positions was evaluated via the end-point Molecular Mechanics-Generalized Born Surface Area (MM-GBSA) approach^29^,^30^,^31^. Compared to the alternative Molecular Mechanics-Poisson Boltzmann Surface Area (MM-PBSA), MM-GBSA is computationally more efficient and has proved to give comparable or even better accuracy in ranking ligand affinities^32^,^33^,^34^. The energy terms were calculated via the program *MMPBSA.py* in AmberTools14^44^. The entropy contribution was not included here, as omission of this term does not qualitatively affect the accurate ranking of a series of ligands against the same receptor^32^,^33^,^34^. The snapshots used for MM-GBSA calculations were extracted since 40 ns of the production simulation at 50-ps intervals, generating 3,600 structures for **S1** to **S4** and 5,600 structures for **S5** and **S6,** respectively (Table 2). In each trajectory snapshot, four water molecules closest to the Mg^2+^ pair were consistently kept and considered as part of the Cas9-sgRNA-dsDNA “receptor”, while the double ions were regarded as a whole “ligand”. Meanwhile, per-residue free energy decomposition was performed to estimate the binding strengths of the RuvC active residues (viz. D10, E762, H983 and D986) to the Mg^2+^ pair. In the final results, we reported the block averaging over even non-overlapping trajectory sections (each containing 400 snapshots) together with the standard error of the mean.

## Additional Information

**How to cite this article**: Zuo, Z. and Liu, J. Cas9-catalyzed DNA Cleavage Generates Staggered Ends: Evidence from Molecular Dynamics Simulations. *Sci. Rep.* **6**, 37584; doi: 10.1038/srep37584 (2016).

**Publisher's note:** Springer Nature remains neutral with regard to jurisdictional claims in published maps and institutional affiliations.

## Supplementary Material

Supplementary Information

## Figures and Tables

**Table 1:** Relative Binding Free Energies via MM-GBSA (kcal/mol).

Simulations	Mean	SEM*
S1	0.0	(8.5)
S2	−2.4	(3.9)
S3	−57.8	(12.4)
S4	−94.7	(16.6)
S5	−181.1	(5.9)
S6	−174.9	(4.3)

^*^Standard error of the mean.

The energy value for **S1** is shifted to zero as the reference point.

**Table 2:** Summary of Each Simulated System.

Simulations	Mg^2+^ in RuvC active center	Mg^2+^ in HNH active center	Solution Mg^2+^ concentration [mM]	Production simulation length [ns]
**S0**	0 ions added	0 ions added	0.0	300
**S1 S2**	2 placed around −3**P**	1 placed	10.0	200
**S3 S4**	2 placed between −4**P** and −3**P**	1 placed	10.0	200
**S5 S6**	2 placed around −4**P**	1 placed	10.0	300

Note that −3**P** and −4**P** denote the phosphate position on the ntDNA 3 and 4 bps upstream of the PAM, respectively.
