# Supplementary material for: Cas9-catalyzed DNA Cleavage Generates Staggered Ends: Evidence from Molecular Dynamics Simulations
Source: Sci Rep. 2016 Nov 22;5:37584. doi: 10.1038/srep37584 (PMC5118739; doi:10.1038/srep37584)
Supplement: Supplementary Information [file srep37584-s1.doc]

Supplementary Information for

Cas9-catalyzed DNA Cleavage Generates Staggered Ends: Evidence from Molecular Dynamics Simulations

Zhicheng Zuo and Jin Liu*****

Department of Pharmaceutical Sciences, University of North Texas Systems College of Pharmacy, University of North Texas Health Science Center, Fort Worth, TX 76107

*****Correspondence author: Jin Liu, [jin.liu@unthsc.edu](mailto:jin.liu@unthsc.edu)

**Table of Contents**

**I. Supplementary Results and Discussion**

**II. Supplementary Figures**

**III. Supplementary Tables**

**IV. Supplementary References**

**I. Supplementary Results and Discussion**

With Mg^2+^ pair placed at -4**P** on the ntDNA, the set of simulations **S5** and **S6** shows homologous results in terms of distance distribution (Figure 2), coordination configuration (Figure 3), binding free energy (Table 1) and Mg^2+^ pair stability (Supplementary Figure S1). Meanwhile, differences are noticed for the remaining simulations sets **S1**&**S2** (Mg^2+^ placed at -3**P**) and **S3**&**S4** (Mg^2+^ pair placed between -3**P** and -4**P**). The distance distributions of **S1** and **S2**, for example, have little overlap with each other (Figure 2D). After inspection of the trajectories, it was found that the Mg^2+^ A in **S1** moved to the 3’-side of -2**P** over one nucleotide (Figure 3A), or even farther toward the HNH domain (Supplementary Figure 2A), resulting in a split distribution for the distance (Figure 2D) and RMSD of the ion pair (Supplementary Figure 1B). The double Mg^2+^ in **S2**, by contrast, were directly bound to the non-bridging oxygen atoms of the successive nucleotides (Supplementary Figure S3A), thereby leading to a shorter distance and formation of a single peak (Figure 2D). Because of the different positioning of the Mg^2+^ pair, **S1** and **S2** also display considerable difference in the separation of the ntDNA backbone and RuvC domain at the catalysis interface (Figure 3A and Supplementary Figure S2 and S3A), as reflected by the distances between the catalytic residues and the opposite phosphates (Figure 2A and 2B). Besides, the binding free energies of **S3** and **S4** are not converged well (Table 1). This discrepancy can be explained by the observation that Asp986 in **S4** forms much stronger interaction with the Mg^2+^ ion than that in **S3** (see Figure 4 and compare Figure 3B and Supplementary S3B).

In order to enhance the reliability of the conclusion drawn in this study, we additionally performed four shorter simulations (4 x 60 ns) on each of the three binding modes with different random seeds. The last 10 ns of the parallel simulations trajectories were used for distance calculations, cluster analysis and binding free energy evaluation as in the main text. The corresponding results are presented in Supplementary Figure S5, Figure S6 and Table S2, respectively. With respect to the fewer longer simulations (2 x 200 or 2 x 300 ns), we did not observe noticeable deviations from the simulations herein, especially for the scenario with Mg^2+^ ions placed at -4**P**. We reason that there likely exist multiple minima on the free energy landscapes underlying the Mg^2+^ pair binding at -3**P** and between -3**P**/-4**P**. It should be noted, on the other hand, that it is difficult to sample all the conformational space with conventional molecular dynamics simulation approach in limited time scale (tens to hundreds of nanoseconds). Furthermore, the current set of Mg^2+^ parameters has not been optimized to fit the experimental exchange rate of the first shell water and thus disfavors the exchange with other coordinating atoms^1,2^, though it represents a best possible compromise targeting the experimental Mg-O distance, hydration free energy, and coordination number^3^. We believe these facts might account for the heterogeneities observed in the simulation sets S**1**/S**2** and S**3**/S**4**. In summary, all the evidence supports the cleavage reaction on the ntDNA occurs at -4**P** through formation of structural and energetically favorable coordination that is most in line with the two-metal-ion mechanism^4-6^.

**II. Supplementary Figures**


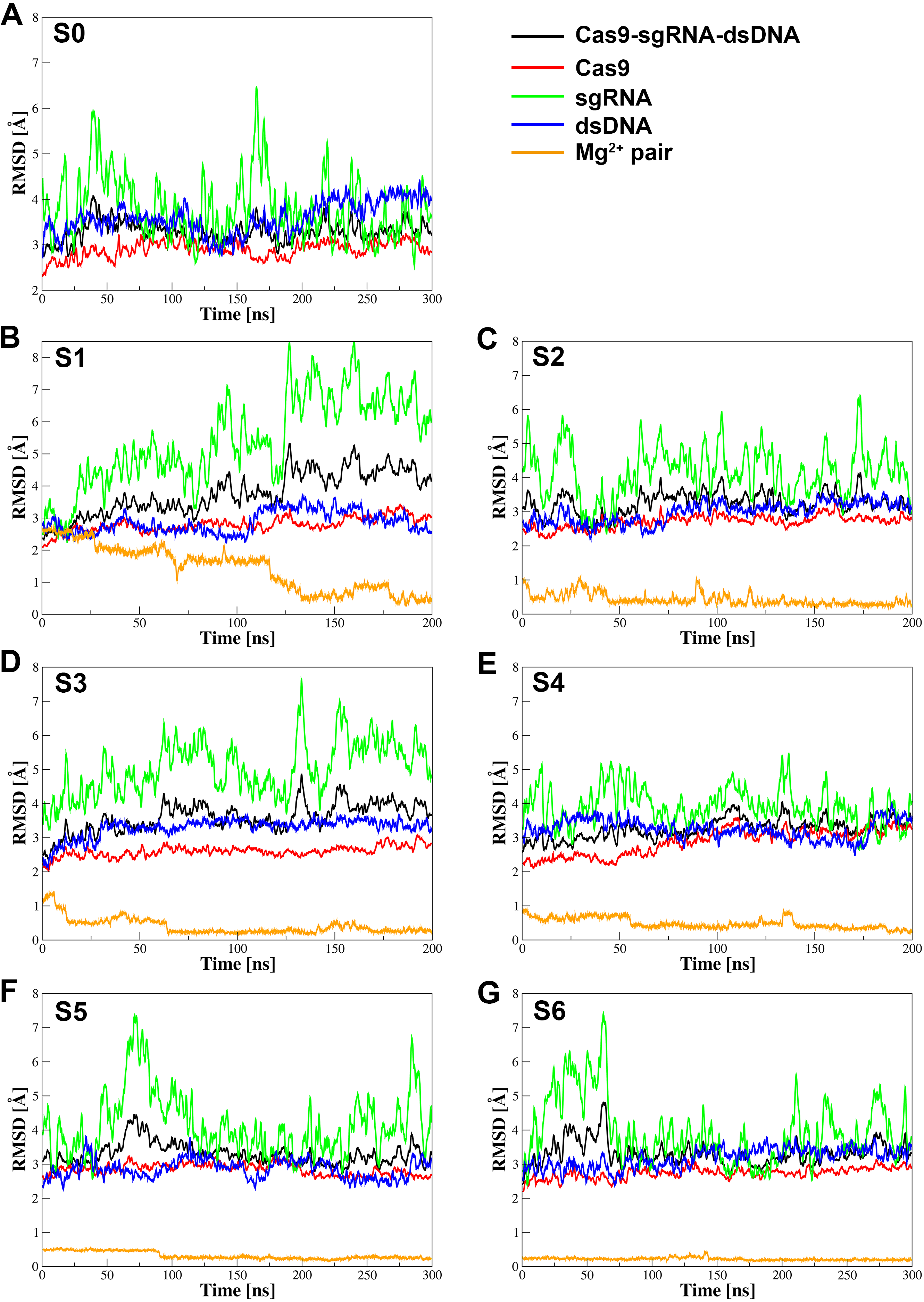


**Figure S1.** Temporal evolution of the backbone root-mean-square deviations (RMSD) of the entire complex (black) and its separate components, Cas9 (red), sgRNA (green) and dsDNA (blue) with reference to the X-ray structure for the simulations **S0** (**A**), **S1** (**B**), **S2** (**C**), **S3** (**D**), **S4** (**E**), **S5** (**F**) and **S6** (**G**). To monitor their mobility, the RMSD of the Mg^2+^ pair (orange) introduced to the RuvC domain was also calculated, by fitting to the catalysis interface (including D10, E762, H983 and D986 on RuvC domain, the nucleotides -3 and -4 on ntDNA and the double Mg^2+^) of the last snapshot in each simulation.


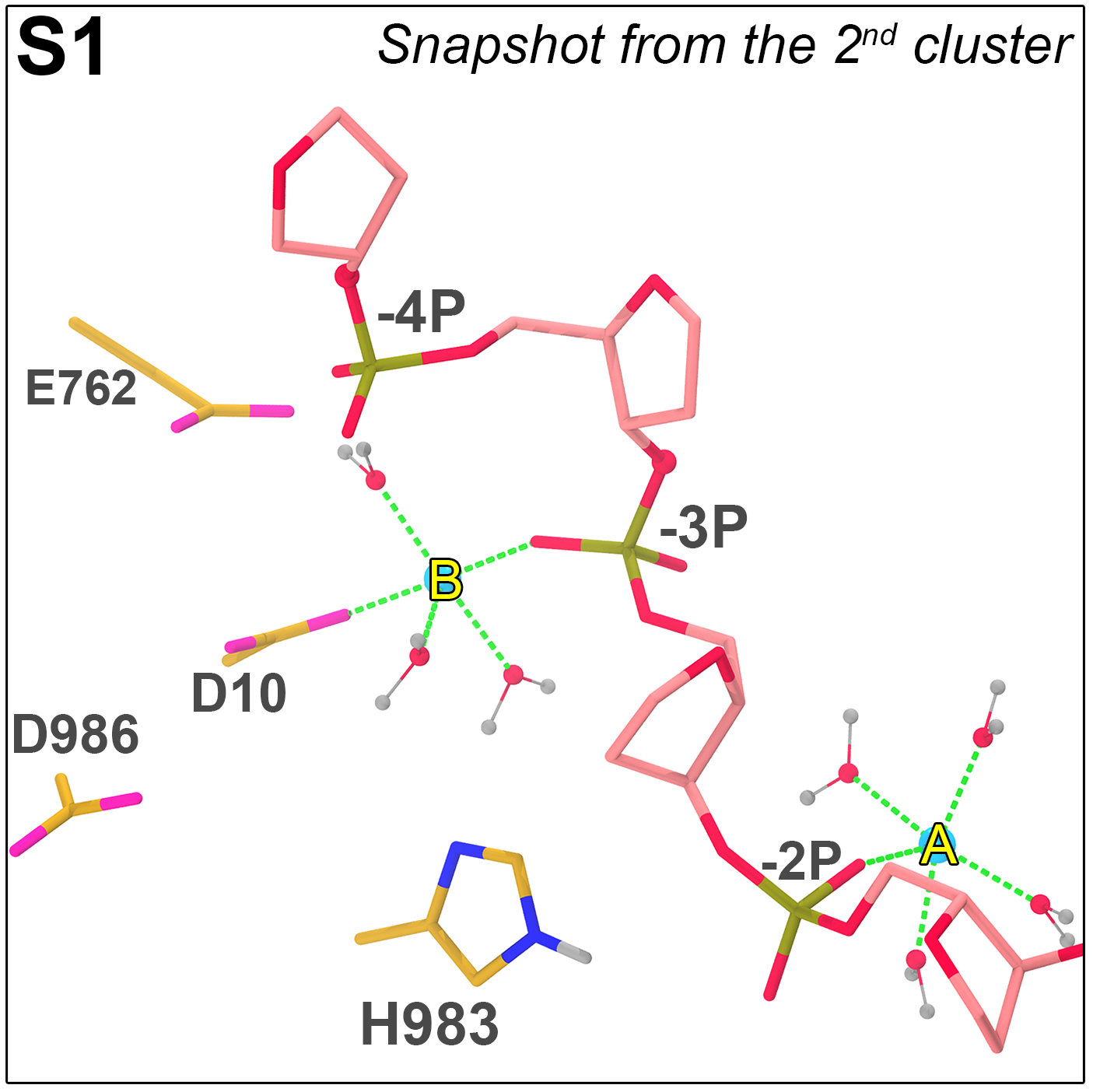


**Figure S2.** The representative coordination configuration involving the Mg^2+^ pair from the second largest cluster of **S1**. See the corresponding snapshot from the first cluster of the same simulation in Figure 3A. The drawing style and coloring scheme are the same to those applied in Figure 3.


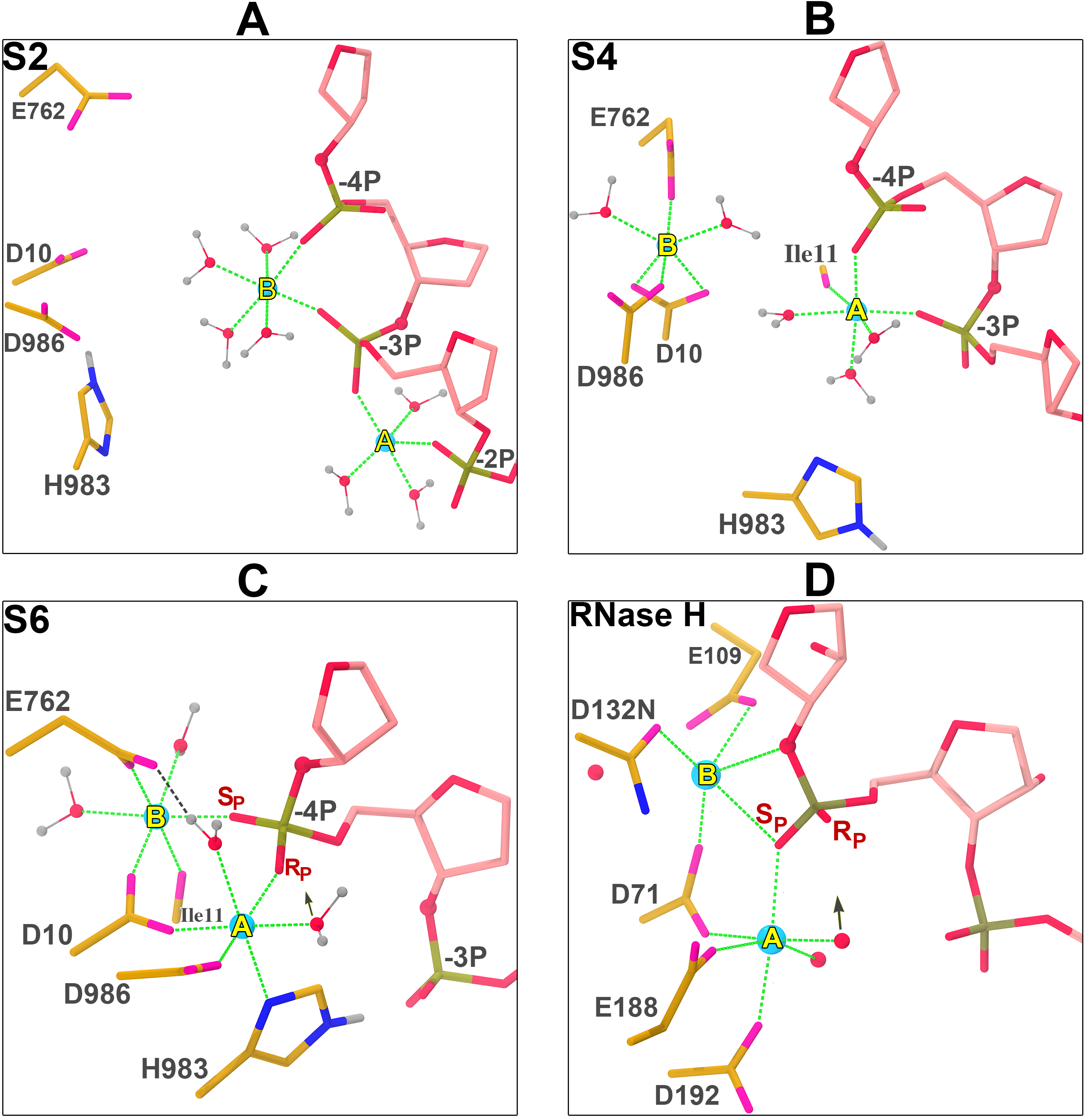


**Figure S3.** The representative coordination configurations involving the Mg^2+^ pair obtained from each set of simulations (A-C) and comparisons with the two-metal-ion catalysis by the RNase H (D). (**A**) The most-populated snapshot from **S2**. (**B**) The most-populated snapshot from **S4**. (**C**) The most-populated snapshot from **S6**. (**D**) Close-up view of the catalytic center of *Bacillus halodurans* RNase H in complex with an RNA/DNA substrate. The RuvC and ntDNA residues are represented in stick model and colored by atom type, and the ligand water are shown as stick and ball style. The Mg^2+^ pair is illustrated as cyan spheres, with the one on the 3’ side of the ntDNA labeled “A” and the other one on the 5’ side labeled “B”. When labeling, the pro-Sp and pro-Rp oxygen atoms of the phosphate group are abbreviated as Sp and Rp, respectively. The green dashed line indicates the coordination bond involving Mg^2+^ (distance < 2.2 Å considered) and the black dashed line denotes the hydrogen bond. The potential nucleophilic water is attached by an arrow. See the other snapshots for **S1**, **S3** and **S5** in Figure 3.


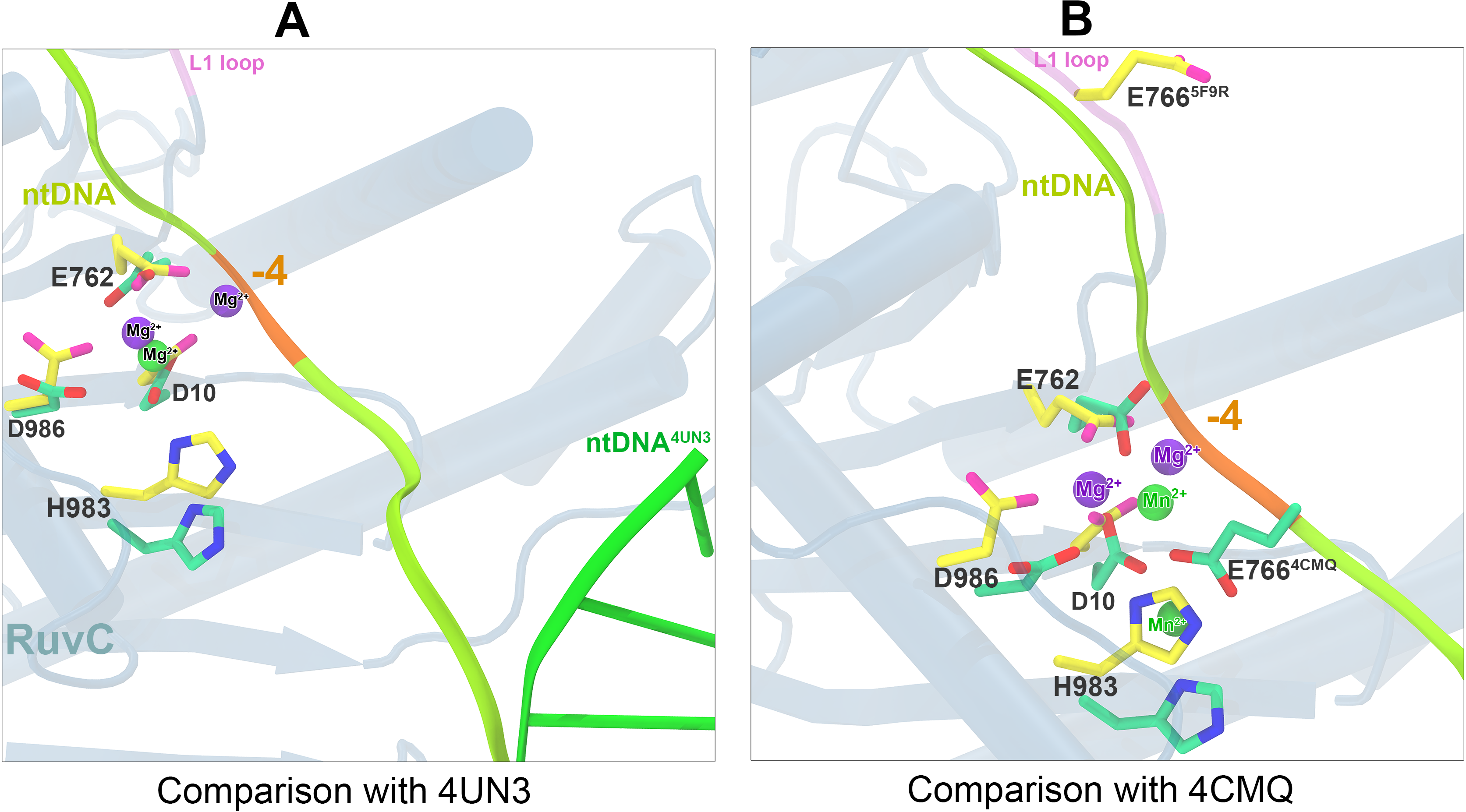


**Figure S4.** Superimposition of the catalytic metal pair captured in the simulation with that in the crystal structure of Mg^2+^-bound Cas9 in complex with sgRNA and incomplete dsDNA (PDB code: 4UN3) (**A**) and that of Mn^2+^(NOT Mg^2+^)-bound Cas9 without nucleic acids (PDB code: 4CMQ) (**B**). The ntDNA in 5F9R is represented as Kelly green cartoon with bases omitted for clarity, in which the nucleotide **-4** is highlighted in orange, and the RuvC domain and the L1-loop (residues 765 to 780) of Cas9 are colored blue and magenta, respectively. The charged residues around the metal ion(s) are shown in the stick model, with those in 5F9R colored yellow and those in 4CMQ and 4UN3 colored cyan. The two Mg^2+^ in the simulation are drawn as violet vdW spheres, and the one Mg^2+^ in 4UN3 and the two Mn^2+^ in 4CMQ as green. It should be mentioned that in 4CMQ, E766 on the L1-loop is engaged to the Mn^2+^ pair, whereas it is shifted to a position far from the catalysis center in 5F9R due to ~180º rigid-body rotation of the HNH domain^7,8^. This observation may partly account for the appreciable difference in metal ion positioning between the simulation snapshot and the X-ray structure (**B**). By contrast, the sole Mg^2+^ in the active center of 4UN3 spatially overlaps with the Mg^2+^ A among the ion pair, leading to similar arrangements of the acidic residue cluster observed in the simulation and 4UN3 (**A**).


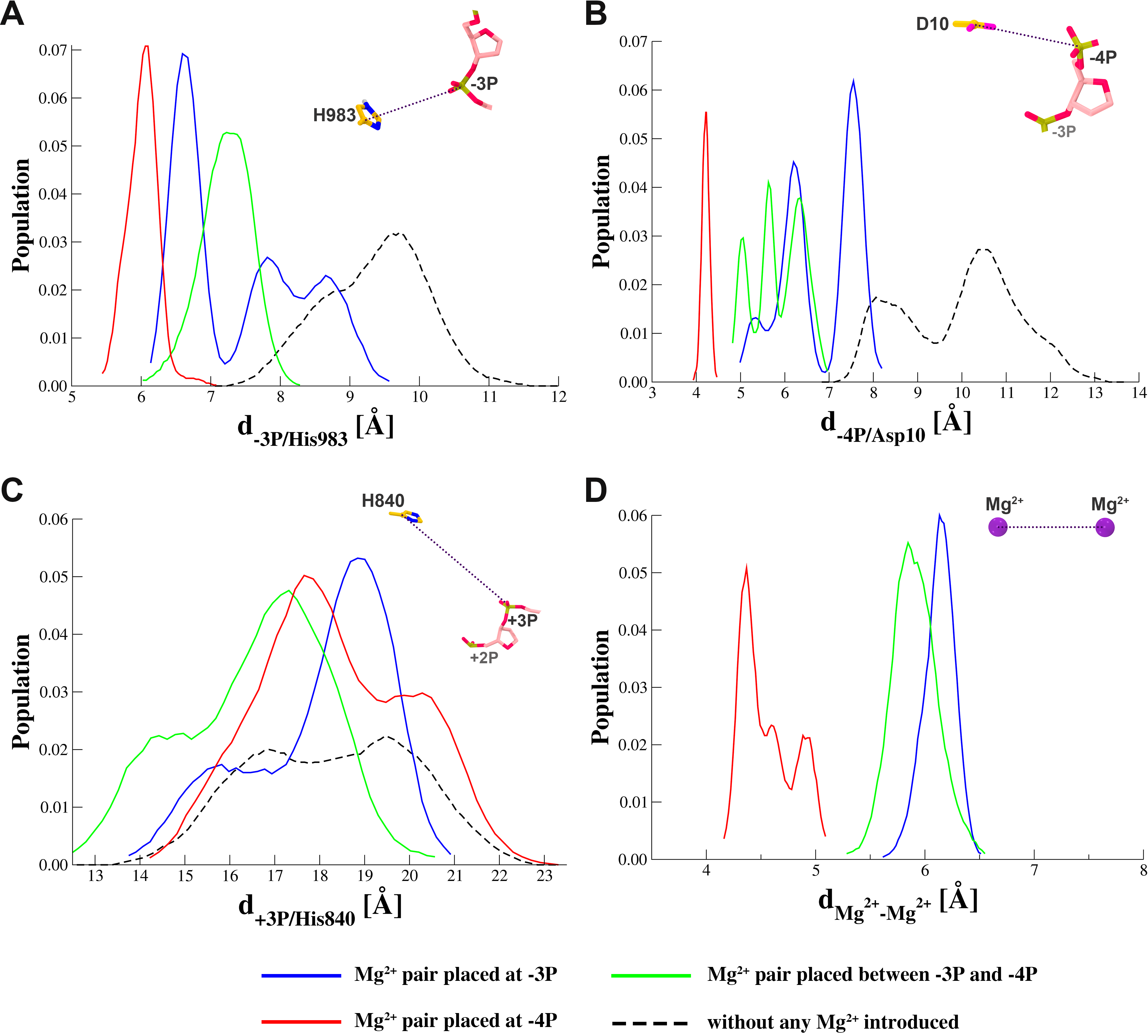


**Figure S5.** Distance distributions between the selected catalytic residues and the opposite phosphates on the target DNA (A-C) and between the two Mg^2+^ ions introduced to the RuvC domain (D) calculated from additional shorter simulations (4 x 60 ns per binding position). The results represent an averaging over the last 10-ns of the four dependent trajectories. This figure is comparable to Figure 2.


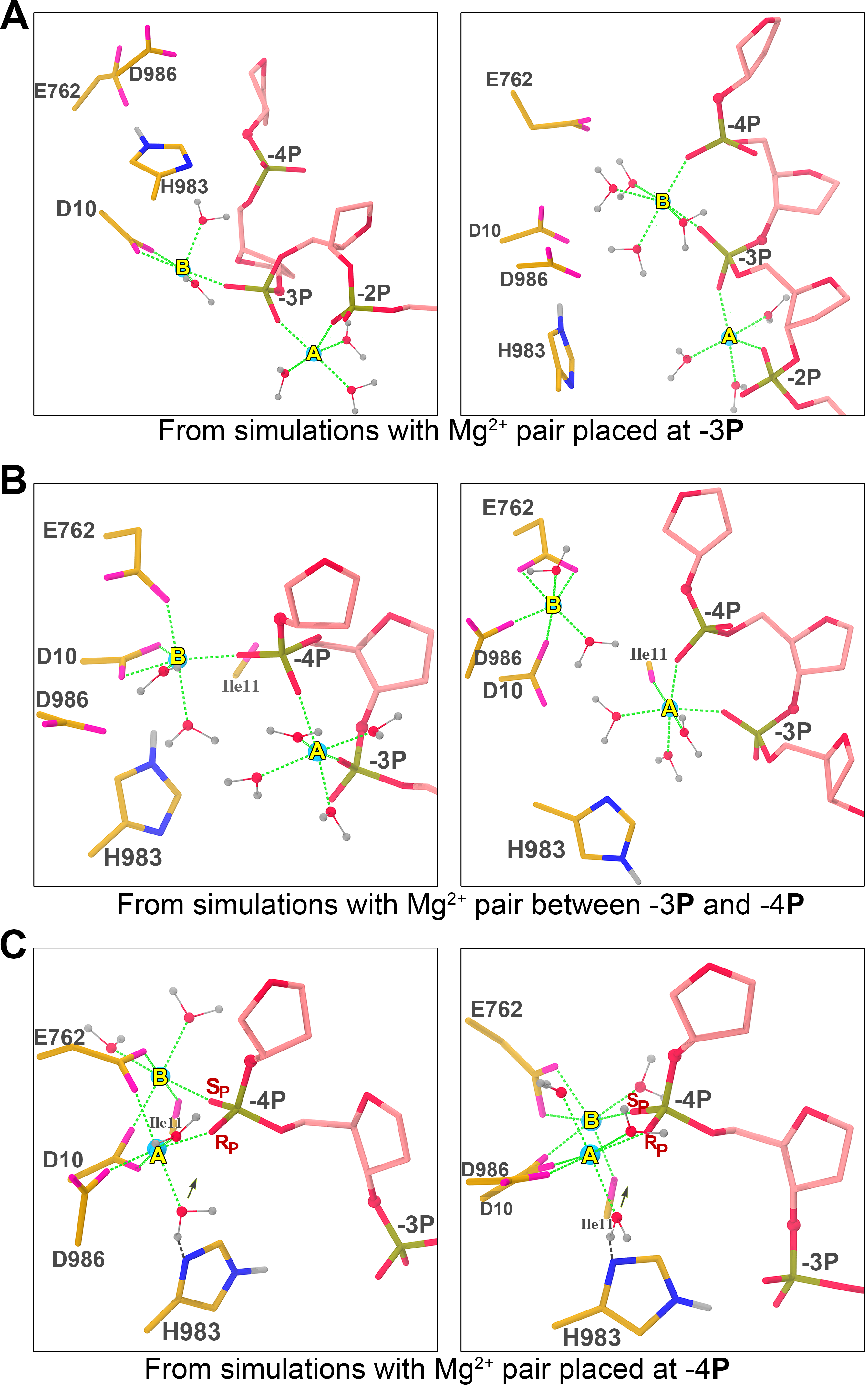


**Figure S6.** The representative coordination configurations involving the Mg^2+^ pair obtained from each set of additional shorter simulations (4 x 60 ns per binding position). The last 10 ns of the four parallel trajectories were aggregated for cluster analysis. This figure is comparable to Figure 3 and Supplementary Figure S3.

**III. Supplementary Tables**

**Table S1.** **Interaction Energy of the Mg^2+^ Pair with Surrounding Residues and Water using a Distance Cutoff of 10 Å (kcal/mol)**

| **Simulations** | Mean | SD |
| --- | --- | --- |
| **S1** | 0.0 | (41.2) |
| **S2** | -182.5 | (32.2) |
| **S3** | -231.5 | (32.9) |
| **S4** | -204.0 | (40.2) |
| **S5** | -421.8 | (30.1) |
| **S6** | -369.2 | (29.6) |

Note that the energy value for **S1** has been shifted to 0.0 kcal/mol as the reference point for the remaining ones.

**Table S2.** **Relative Binding Free Energies via MM-GBSA approach (kcal/mol)a**

| **Simulations** | Mean | SD | Mean | SEM |
| --- | --- | --- | --- | --- |
| **-3P^b^** | -9.6 | (9.0) | 0.0 | (6.1) |
|  | -6.6 | (6.9) |  |  |
|  | 0.0 | (7.7) |  |  |
|  | -14.62 | (9.4) |  |  |
| **-3P/-4P^c^** | -62.4 | (7.5) | -74.6 | (14.7) |
|  | -80.4 | (14.8) |  |  |
|  | -94.9 | (9.3) |  |  |
|  | -91.4 | (8.8) |  |  |
| **-4P^d^** | -184.2 | (11.4) | -165.7 | (7.4) |
|  | -169.6 | (10.0) |  |  |
|  | -167.5 | (11.0) |  |  |
|  | -172.1 | (10.7) |  |  |

^a^ This Table is calculated from the supplemental shorter simulations (4 x 60 ns per binding pose) and comparable to Table 1.

**^b^** The simulations with Mg^2+^ pair placed at -3**P**

c The simulations with Mg^2+^ pair placed between -3**P**/-4**P**

d The simulations with Mg^2+^ pair placed at -4**P**.

**IV. Supplementary References**

1. Panteva, M. T., Giambasu, G. M. & York, D. M. Comparison of Structural,

Thermodynamic, Kinetic and Mass Transport Properties of Mg2+ Ion Models

Commonly used in Biomolecular Simulations. *J. Comput. Chem.* **36**, 970-982 (2015).

2. Allner, O., Nilsson, L. & Villa, A. Magnesium Ion-Water Coordination and Exchange

in Biomolecular Simulations. *J. Chem. Theory Comput.* **8**, 1493-1502 (2012).

3. Li, P., Roberts, B. P., Chakravorty, D. K. & Merz, K. M., Jr. Rational Design of

Particle Mesh Ewald Compatible Lennard-Jones Parameters for +2 Metal Cations in

Explicit Solvent. *J. Chem. Theory Comput.* **9**, 2733-2748 (2013).

4. Yang, W., Lee, J. Y. & Nowotny, M. Making and breaking nucleic acids: two-Mg2+-

ion catalysis and substrate specificity. *Mol. Cell* **22**, 5-13 (2006).

5. Yang, W. An equivalent metal ion in one- and two-metal-ion catalysis. *Nat. Struct.*

*Mol. Biol*. **15**, 1228-1231 (2008).

6. Yang, W. Nucleases: diversity of structure, function and mechanism. *Q. Rev. Biophys*.

**44**, 1-93 (2011).

7. Jinek, M. *et al.* Structures of Cas9 endonucleases reveal RNA-mediated

conformational activation. *Science* **343**, 1247997 (2014).

8. Jiang, F. G. *et al.* Structures of a CRISPR-Cas9 R-loop complex primed for DNA

cleavage. *Science* **351**, 867-871 (2016).
